# Frequency of adding salt to foods, metabolic signature, and the risk of extracoronary atherosclerotic vascular disease progression

**DOI:** 10.3389/fnut.2026.1870553

**Published:** 2026-07-24

**Authors:** Cheng Wei, Chenghui Cao, Wen Gong, Bilian Yu, Liang Tang, Zhaowei Zhu, Shenghua Zhou

**Affiliations:** 1 Department of Cardiovascular Medicine, The Second Xiangya Hospital, Central South University, Changsha, Hunan, China; 2 Key Laboratory of Cardiometabolic Medicine in Hunan, Changsha, Hunan, China; 3 Department of Cardiology, Erasmus MC, University Medical Center Rotterdam, Rotterdam, Netherlands; 4 Xiangya School of Medicine, Central South University, Changsha, Hunan, China; 5 Libin Cardiovascular Institute of Alberta, University of Calgary, Health Sciences Centre, Calgary, AB, Canada; 6 Research Institute of Blood Lipid and Atherosclerosis, Central South University, Changsha, Hunan, China

**Keywords:** adding salt to foods, carotid artery stenosis, extracoronary atherosclerotic vascular disease, metabolic signature, peripheral artery disease

## Abstract

**Background:**

The frequency of adding salt to foods reflects an individual’s long-term salt preference, and excessive salt intake may increase cardiovascular risk. However, the association between this behavioral indicator and extracoronary atherosclerotic vascular disease, along with its underlying metabolic signature, remains inadequately characterized.

**Methods:**

We included 495,291 participants from the UK Biobank and assessed the frequency of adding salt to foods. Cox proportional hazards regressions modeled the associations of salt-adding frequency with incident peripheral arterial disease (PAD) and incident carotid artery stenosis (CAS), and related events, respectively.

**Results:**

During a median of 13.9 years of follow-up, 7,461 incident cases of PAD and 8,067 cases of CAS were identified. When fully adjusted for confounders, participants who sometimes, usually, or always added salt to foods showed an increased risk of developing PAD of 8%, 16%, and 35%, respectively, compared to those who never/rarely did. A comparable pattern of risk increase was also evident for CAS, with 26% higher risk in participants who always added salt to foods. In addition, consistent associations were observed for vascular-related events. Genetic analyses further revealed that the association between salt-adding frequency and PAD was independent of genetic predisposition. Finally, a metabolic signature for salt-adding frequency was constructed using 26 metabolites, which mediated 36.4% and 19.9% of the increased risks for PAD and CAS, respectively.

**Conclusion:**

Our study shows that frequently adding salt to foods is associated with an increased risk of extracoronary atherosclerotic vascular disease and related events, with circulating metabolites playing a partial mediating role.

## 1 Introduction

Extracoronary atherosclerotic vascular disease, particularly peripheral artery disease (PAD) and carotid artery stenosis (CAS), are emerging as significant global public health challenges, affecting hundreds of millions worldwide (1). Current estimates indicate that over 200 million individuals suffer from PAD, with incidence markedly increasing with age and higher prevalence (2). Furthermore, clinically significant CAS affects approximately 5%–10% of people over 70 years old and remains a major contributor to ischemic stroke (3). Despite their well-established links to myocardial infarction, stroke, and mortality (1), these extracoronary vascular outcomes are often under-recognized and underdiagnosed due to their largely asymptomatic early stages and lack of routine screening (4, 5). Systematic investigations into their pathophysiology and biological drivers remain limited, underscoring the urgent need for further research.

Although the link between high sodium intake and hypertension is well established (6), its association with extracoronary vascular outcomes remains understudied. First, most previous studies have focused on total sodium intake, typically assessed through dietary questionnaires or urinary sodium excretion (7–9). The frequency of adding salt to foods shows a positive association with estimated 24-h urinary sodium excretion (10). This association reflects a person’s long-term preference for salty taste and also supports the reliability of this measure (11). Compared with the gold standard, which is the average of multiple non-consecutive 24-h urine collections, this measure may be easier to use for assessing salt habits in large population studies. Second, existing research is often limited to structural vascular markers, with small sample sizes and a lack of large-scale prospective studies providing systematic evaluation (7–9). Third, while some studies have explored the association between salt-adding frequency and stroke risk (11, 12), none have examined the progression from extracoronary atherosclerotic vascular disease to stroke or major adverse limb events, leaving the natural history of disease underexplored. Recent prospective evidence suggests that lower frequency of salt addition is associated with reduced risks of cardiovascular disease (CVD) (11), atrial fibrillation (AF) (13), and type 2 diabetes mellitus (14), potentially mediated through blood pressure (9). However, the interpretation underlying these associations remain incompletely understood. Circulating metabolites have emerged as important intermediaries linking dietary behaviors to disease risk (15). Metabolomic approaches can be employed to identify systemic metabolic alterations induced by sodium intake, which may provide insights into the biological pathways linked to extracoronary vascular outcomes.

Therefore, in this study, we utilized prospective cohort data from the UK Biobank to systematically evaluate the association between the frequency of adding salt to food and the risk of extracoronary atherosclerotic vascular disease and related events. Furthermore, we explored the potential roles of genetic factors and circulating metabolites in these associations.

## 2 Materials and methods

### 2.1 Study population

Between 2006 and 2010, the UK Biobank initiative recruited over 500,000 participants from 22 assessment centers across England, Scotland, and Wales, establishing a large prospective cohort (16). The study design and data collection procedures have been detailed in prior publications (17). All participants provided written informed consent, and the study was approved by the North West Multi-Centre Research Ethics Committee (11/NW/0382). From the initial sample of 501,969 individuals, we excluded those with missing data on the baseline frequency of adding salt to foods (*n* = 1,125), those with prevalent PAD (*n* = 2,315) or CAS (*n* = 1,964) at baseline, and those who were lost to follow-up (*n* = 1,274). After these exclusions, 495,291 participants remained eligible for the primary analyses (Supplementary Figure 1).

### 2.2 Assessment of frequency of adding salt at the table

Information on dietary habits was collected at baseline using a touchscreen questionnaire. To determine how frequently participants added salt to their food, they were asked: “Do you add salt to your foods? (Do not include salt used in cooking).” Participants selected from one of the following five options: (1) never/rarely; (2) sometimes; (3) usually; (4) always; or (5) prefer not to answer. Individuals who selected “prefer not to answer” were treated as missing data and subsequently excluded from the analysis. To assess dietary habits, participants were asked, “Have you made any major changes to your diet in the last 5 years?” in the baseline questionnaire. They chose one of the following options: (1) no; (2) yes, because of illness; (3) yes, because of other reasons; or (4) prefer not to answer. The validity of the questionnaire assessing the frequency of adding salt at the table has been confirmed in multiple studies (10, 11).

### 2.3 Assessment of extracoronary atherosclerotic vascular disease

Incident cases of PAD or CAS were identified using corresponding the 10th revision of the International Classification of Diseases (ICD-10) and Office of Population Censuses and Surveys versions 4 (OPCS-4). Deaths among patients with PAD/CAS were ascertained through linkage with the death registry. Follow-up time was calculated from the baseline assessment date until the first occurrence of any of the following: diagnosis of PAD/CAS, death, or the censoring date (December 31, 2022). Prevalent cases of PAD and CAS (with onset at or before baseline) were identified based on the above criteria as well as on relevant self-reported history. In line with previous research (18, 19), major adverse limb events (MALE) and ischemic stroke were selected as clinically relevant endpoints to assess disease progression in PAD and CAS, respectively. Detailed definitions for these outcomes are provided in Supplementary Table 1.

### 2.4 Assessment of covariates

According to prior studies (18–20), potential confounding variables were identified, classified as sociodemographic characteristics, lifestyle factors, comorbidities, and medication use. Sociodemographic characteristics were obtained at baseline via self-report or touch-screen questionnaires and comprised age, sex (female or male), ethnicity (non-White or White), Townsend deprivation index (TDI), body mass index [BMI, (<30 kg/m^2^ or ≥30 kg/m^2^)], and education level (College/University or others). The TDI incorporates diverse indicators related to social class, employment, and housing conditions, serving as an area-based measure of socioeconomic status, where higher scores correspond to increased levels of deprivation. Lifestyle factors considered for adjustment included smoking status (never, previous, or current), alcohol intake frequency (<3 times/week or ≥3 times/week), physical activity (<150 min/week or ≥150 min/week), sleep duration (short, normal, or long), and diet score (<3 points or ≥3 points). Physical activity was categorized according to established guidelines (21), which recommend at least 150 min of moderate activity, 75 min of vigorous activity, or an equivalent combination weekly. Estimated glomerular filtration rate (eGFR) was estimated using the 2021 race-free Chronic Kidney Disease Epidemiology Collaboration (CKD-EPI) equations based on creatinine and cystatin C (22). A diet score ranging from 0 to 5 was constructed based on five previously reported dietary components (23) to reflect overall dietary quality (Supplementary Table 2). Comorbidities, including dyslipidemia, diabetes, hypertension, CVD (encompassing coronary heart disease, stroke, and heart failure), AF, and chronic kidney disease, were documented as present or absent based on self-report and medical records. Medication use covered aspirin, lipid-lowering drugs, diabetes medications, and antihypertensive agents. A summary of missing covariate data is presented in Supplementary Table 3.

### 2.5 Assessment of genetic susceptibility

Due to limitations in existing literature, genetic susceptibility was assessed only for PAD. A polygenic risk score (PRS) for PAD was constructed using 19 independent single-nucleotide polymorphisms (SNPs) identified as significantly associated with PAD in genome-wide association study (24) (Supplementary Table 4). The score was calculated by a weighted method (25), and validation of these SNPs have been described in previous publications (20). Based on the distribution of the PRS, participants were stratified into low (quintile 1), intermediate (quintiles 2–4), and high (quintile 5) genetic risk groups.

### 2.6 Metabolomic profiling

Metabolomic profiling was performed on baseline plasma samples from approximately 280,000 UK Biobank participants using a high-throughput nuclear magnetic resonance platform. Detailed protocols for sample handling and metabolite quantification have been published previously (26). This assay quantified 251 metabolites, encompassing lipoprotein lipids, fatty acids, and various low-molecular-weight metabolites. To make the data approximate normal distributions and standardize the values, all metabolites underwent natural logarithm transformation followed by Z-score transformation prior to analysis.

### 2.7 Statistical analyses

Baseline characteristics were summarized according to the frequency of adding salt to foods. Continuous variables are presented as median (interquartile range, IQR), and categorical variables as number (percentage).

Cumulative risk curves were plotted by the frequency of adding salt to foods using Kaplan-Meier methods. The associations of salt addition frequency with the risks of PAD, CAS, and their related events were evaluated using multivariable Cox proportional hazards models. Results are reported as hazard ratios (HRs) with 95% confidence intervals (CIs). We assessed the proportional hazards assumption using Schoenfeld residuals and added time-interaction terms for covariates that violated the assumption. Three analytical models were constructed: Model 1 adjusted for age, sex, and ethnicity; Model 2 further adjusted TDI, education level, BMI, smoking status, alcohol intake frequency, sleep duration, physical activity, hypertension, dyslipidemia, diabetes, CVD, and aspirin use; Model 3 additionally adjusted eGFR, diet score, AF, CKD, antihypertensive medication, lipid-lowering medication, and diabetes medications. To minimize potential over-adjustment, Models 1 and 2 were treated as the primary analyses, while Model 3 was used for sensitivity analysis. For missing data, continuous covariates were imputed with median values, and categorical covariates were handled using a missing indicator category. In genetic analyses, additional adjustments were made for genotype measurement batch and the first ten principal components of genetic ancestry. Furthermore, we evaluated the associations between salt-adding frequency and PAD risk within each genetic risk stratum.

To construct a metabolic signature for salt-adding frequency, we applied least absolute shrinkage and selection operator (LASSO) regression to the 251 standardized plasma metabolites. The model treated the frequency of adding salt (always vs. never/rarely) as the response variable. The optimal lambda parameter was determined through 10-fold cross-validation, selecting the value within one standard error of the minimum cross-validated error. The metabolic signature score was constructed as a weighted sum of the selected metabolites, with weights derived from LASSO regression coefficients. We investigated the effects of frequent salt addition (always vs. never/rarely) on the metabolic signature score and individual metabolites using multivariable linear regression. The associations of this score and of individual metabolites with PAD and CAS were evaluated using multivariable Cox proportional hazards models. Additionally, restricted cubic splines with three knots (at the 5th, 50th, and 95th percentiles) were employed to examine nonlinear relationships. Finally, we performed mediation analysis using the R package CMAverse (version 0.1.0) to assess whether the metabolic signature score or specific metabolites mediate the association between frequency of adding salt to foods and the study outcomes.

Stratified analyses were conducted according to age (<60 or ≥60 years), sex (female or male), BMI (<30 or ≥30 kg/m^2^), smoking status (non-current or current), alcohol intake frequency (<3 or ≥3 times/week), dyslipidemia (no or yes), diabetes (no or yes), CVD (no or yes), and hypertension (no or yes). Interaction terms between salt-adding frequency and each stratification variable were incorporated into the Cox models, and their significance was assessed using the Wald test. To evaluate the robustness of the primary findings, we performed a series of sensitivity analyses. First, we excluded participants with missing covariate values, which accounted for less than 10% of the cohort. Second, the analysis was repeated after removing individuals with prevalent CVD at baseline. Third, to address potential reverse causality, we excluded participants who developed PAD or CAS within the initial 2 years of follow-up. Fourth, we further restricted the analysis to those who reported no major dietary changes in the past 5 years. Finally, further adjusting for all covariates included in Model 3.

All analyses were conducted with R (version 4.2.2), with statistical significance defined as a two-sided *P* < 0.05.

## 3 Results

### 3.1 Population characteristics

A total of 495,291 participants were included in this study. Baseline characteristics, stratified by the frequency of adding salt to foods, are presented in Table 1. Compared to those who seldom added salt, participants in the high-frequency group were more likely to be male, of non-White ethnicity, and to have a higher TDI and BMI. They also exhibited a lower educational attainment and a higher prevalence of CVD. Additionally, this group was characterized by a cluster of less healthy lifestyle behaviors, including a higher proportion of current smokers, physical inactivity, suboptimal sleep patterns, and a lower diet score.

**TABLE 1:** Baseline characteristics of participants based on the frequency of adding salt to foods in the UK Biobank.

Characteristic	Frequency of adding salt to foods			
	Never/rarely	Sometimes	Usually	Always
**Participants, *n***	274,795	138,890	57,620	23,986
**Age in years, median [IQR]**	58.0 (50.0, 63.0)	58.0 (50.0, 63.0)	58.0 (51.0, 64.0)	57.0 (49.0, 63.0)
**Sex, *n* (%)**				
Female	154,466 (56%)	75,274 (54%)	28,256 (49%)	12,417 (52%)
Male	120,329 (44%)	63,616 (46%)	29,364 (51%)	11,569 (48%)
**White ethnicity, *n* (%)**	262,420 (95%)	129,678 (93%)	53,810 (93%)	20,995 (88%)
**TDI, median [IQR]**	−2.3 (−3.7, 0.2)	−2.1 (−3.6, 0.7)	−1.9 (−3.5, 0.9)	−0.9 (−3.1, 2.5)
**eGFR, median [IQR] ml/min/1.73 m^2^**	96.3 (86.9, 105.4)	95.8 (86.4, 104.9)	95.1 (85.7, 104.3)	95.0 (85.5, 104.6)
**BMI, *n* (%)**				
<30 kg/m^2^	212,877 (77%)	103,307 (74%)	42,035 (73%)	16,995 (71%)
≥30 kg/m^2^	61,918 (23%)	35,583 (26%)	15,585 (27%)	6,991 (29%)
**Education, *n* (%)**				
College or university	94,436 (34%)	43,171 (31%)	17,204 (30%)	4,807 (20%)
Others	175,855 (64%)	93,007 (67%)	39,309 (68%)	18,539 (77%)
Unknown	4,504 (1.6%)	2,712 (2.0%)	1,107 (1.9%)	640 (2.7%)
**Smoking status, *n* (%)**				
Never	162,885 (59%)	72,786 (52%)	26,038 (45%)	9,672 (40%)
Previous	89,129 (32%)	49,946 (36%)	22,617 (39%)	8,567 (36%)
Current	21,865 (8.0%)	15,584 (11%)	8,729 (15%)	5,602 (23%)
Unknown	916 (0.3%)	574 (0.4%)	236 (0.4%)	145 (0.6%)
**Alcohol intake frequency, *n* (%)**				
<3 times/week	159,594 (58%)	76,931 (55%)	29,785 (52%)	13,761 (57%)
≥3 times/week	115,025 (42%)	61,815 (45%)	27,762 (48%)	10,174 (42%)
Unknown	176 (<0.1%)	144 (0.1%)	73 (0.1%)	51 (0.2%)
**Physical activity, *n* (%)**				
<150 min/week	39,570 (14%)	19,655 (14%)	9,170 (16%)	4,266 (18%)
≥150 min/week	175,164 (64%)	85,530 (62%)	34,682 (60%)	12,738 (53%)
Unknown	60,061 (22%)	33,705 (24%)	13,768 (24%)	6,982 (29%)
**Sleep duration, *n* (%)**				
Normal, 7–8 h/d	188,471 (69%)	93,074 (67%)	37,631 (65%)	13,865 (58%)
Short, <7 h/d	64,693 (24%)	34,442 (25%)	15,013 (26%)	7,399 (31%)
Long, >8 h/d	20,155 (7.3%)	10,434 (7.5%)	4,594 (8.0%)	2,414 (10%)
Unknown	1,476 (0.5%)	940 (0.7%)	382 (0.7%)	308 (1.3%)
**Diet score, *n* (%)**				
<3	129,236 (47%)	67,382 (49%)	29,102 (51%)	13,218 (55%)
≥3	145,559 (53%)	71,508 (51%)	28,518 (49%)	10,768 (45%)
**Comorbidities, *n* (%)**				
Dyslipidemia	41,024 (15%)	20,032 (14%)	8,658 (15%)	3,434 (14%)
Diabetes	7,057 (2.6%)	3,920 (2.8%)	1,567 (2.7%)	666 (2.8%)
Hypertension	76,493 (28%)	35,008 (25%)	14,174 (25%)	5,885 (25%)
CVD	13,820 (5.0%)	6,694 (4.8%)	3,045 (5.3%)	1,418 (5.9%)
AF	4,468 (1.6%)	2,198 (1.6%)	966 (1.7%)	386 (1.6%)
CKD	3,389 (1.2%)	1,519 (1.1%)	602 (1.0%)	254 (1.1%)
**Medication, *n* (%)**				
Aspirin	36,888 (13%)	18,471 (13%)	8,134 (14%)	3,345 (14%)
Lipid-lowering medication	47,049 (17%)	22,862 (16%)	9,829 (17%)	3,893 (16%)
Diabetes medication	9,829 (3.6%)	5,367 (3.9%)	2,161 (3.8%)	941 (3.9%)
Antihypertensive medication	59,568 (22%)	26,517 (19%)	10,700 (19%)	4,321 (18%)

IQR, interquartile range; TDI, Townsend deprivation index; BMI, body mass index; CVD, cardiovascular disease; AF, atrial fibrillation; CKD, chronic kidney disease. Categorical variables are presented as numbers (percentages). Continuous variables are presented as median (interquartile range).

### 3.2 Frequency of adding salt to foods and risk of incident and progressive extracoronary vascular disease

**FIGURE 1:**
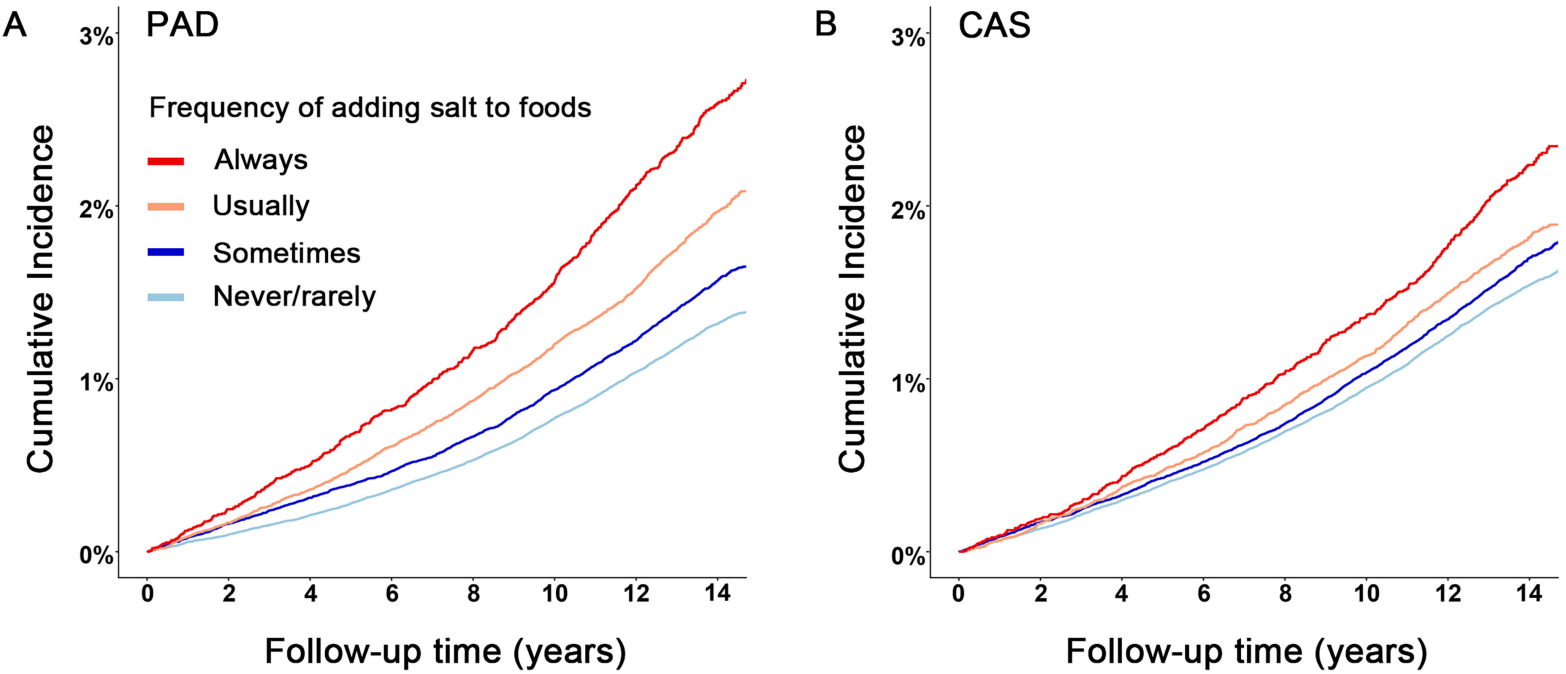
Kaplan-Meier curves showing the cumulative incidence of **(A)** peripheral artery disease and **(B)** carotid artery stenosis according to the frequency of adding salt to foods.

Over a median 13.9-year follow-up, 7,461 incident PAD and 8,067 CAS events were recorded (Supplementary Table 5). The Kaplan-Meier curves demonstrated significant separation in cumulative risk by salt-adding frequency for both conditions (Figure 1). In the fully adjusted model (Model 2), a higher frequency of adding salt to foods was significantly associated with an increased risk of both PAD and CAS (Table 2). Compared to the reference group (never/rarely), the multivariable-adjusted HRs (95% CIs) for PAD showed a graded increase: 1.08 (1.02–1.14) for those who sometimes added salt, 1.16 (1.09–1.24) for those who usually added salt, and 1.35 (1.24–1.48) for those who always added salt. A comparable pattern of risk increase was also evident for CAS, with HRs (95% CIs) of 1.07 (1.02–1.13), 1.06 (0.99–1.13), and 1.26 (1.15–1.38) across the same categories (both *P* for trend < 0.001). We further assessed whether this habit was associated with major complications in patients with established vascular disease. Of the 7,461 PAD cases, 809 developed MALE; of the 8,067 CAS cases, 3,275 progressed to ischemic stroke. A consistently elevated risk was observed between higher salt-addition frequency and these complications (Table 2). Specifically, compared to the reference group, for MALE, the HRs (95% CIs) were 1.20 (1.02–1.41), 1.26 (1.02–1.54), and 1.68 (1.32–2.14) for the sometimes, usually, and always groups, respectively. For ischemic stroke, the corresponding HRs (95% CIs) were 1.10 (1.02–1.19), 1.03 (0.93–1.15), and 1.26 (1.09–1.45) (both *P* for trend < 0.01).

**TABLE 2:** Association between the frequency of adding salt to foods and risk of incident extracoronary vascular disease and related events.

Model	Frequency of adding salt to foods, HR (95% CI)				*P* for trend
	Never/rarely	Sometimes	Usually	Always	
Incident PAD					
Cases/N	3,575/274,795	2,152/138,890	1,123/57,620	611/23,986	
Model 1	1.00 (reference)	1.18 (1.12–1.25)	1.36 (1.27–1.46)	1.96 (1.80–2.14)	<0.001
Model 2	1.00 (reference)	1.08 (1.02–1.14)	1.16 (1.09–1.24)	1.35 (1.24–1.48)	<0.001
PAD progression to MALE					
Cases/N	350/3,575	245/2,152	129/1,123	85/611	
Model 1	1.00 (reference)	1.37 (1.16–1.61)	1.58 (1.29–1.93)	2.73 (2.15–3.46)	<0.001
Model 2	1.00 (reference)	1.20 (1.02–1.41)	1.26 (1.02–1.54)	1.68 (1.32–2.14)	<0.001
Incident CAS					
Cases/N	4,191/274,795	2,314/138,890	1,034/57,620	528/23,986	
Model 1	1.00 (reference)	1.08 (1.03–1.14)	1.09 (1.02–1.16)	1.44 (1.32–1.58)	<0.001
Model 2	1.00 (reference)	1.07 (1.02–1.13)	1.06 (0.99–1.13)	1.26 (1.15–1.38)	<0.001
CAS progression to stroke					
Cases/N	1,672/4,191	962/2,314	414/1,034	227/528	
Model 1	1.00 (reference)	1.13 (1.04–1.22)	1.08 (0.97–1.21)	1.54 (1.34–1.77)	<0.001
Model 2	1.00 (reference)	1.10 (1.02–1.19)	1.03 (0.93–1.15)	1.26 (1.09–1.45)	0.005

PAD, peripheral artery disease; CAS, carotid artery stenosis; MALE, major adverse limb events. Model 1: Adjusted for age, sex, ethnicity. Model 2: further adjusted for Townsend deprivation index, education, body mass index, smoking status, alcohol intake frequency, sleep duration, physical activity, hypertension, dyslipidemia, diabetes, cardiovascular disease, and aspirin use.

### 3.3 Joint associations of the frequency of adding salt to foods and genetic susceptibility with PAD risk

**FIGURE 2:**
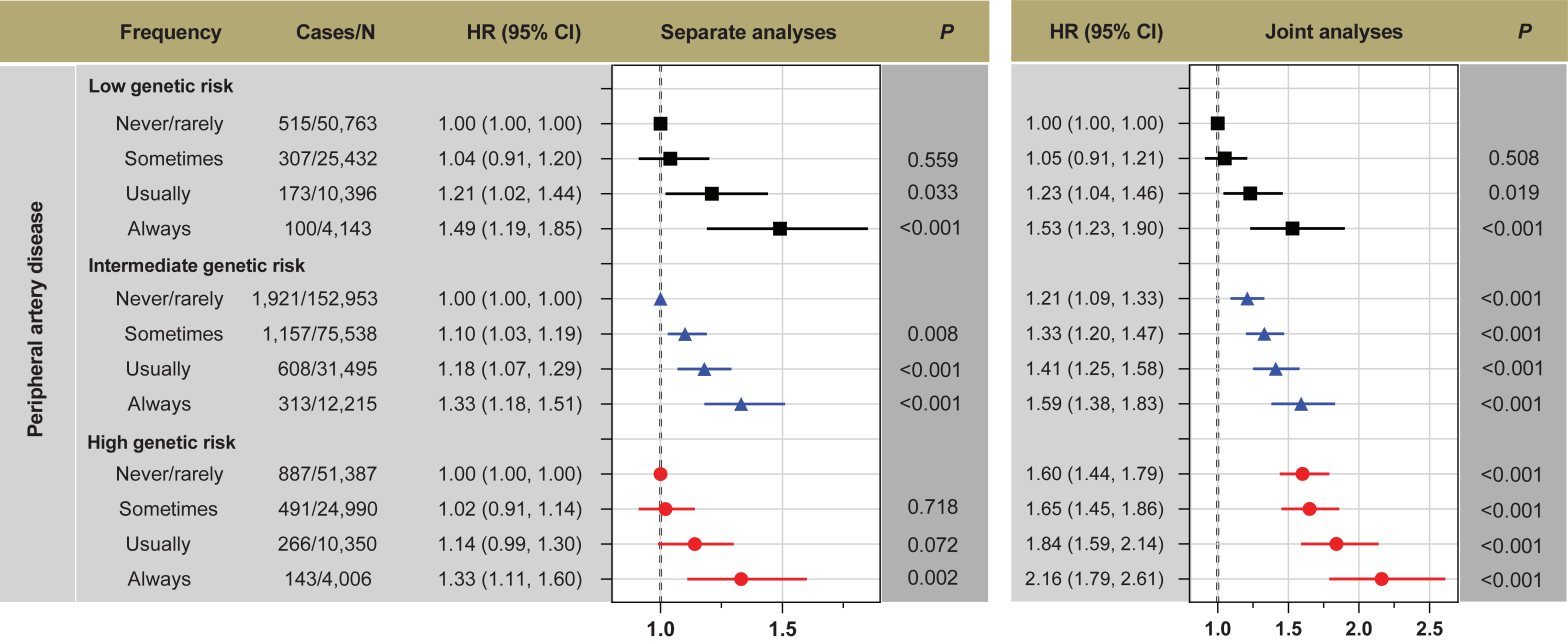
Association between the frequency of adding salt to foods and peripheral artery disease risk across genetic risk strata. *P* for interaction between salt-adding frequency and genetic risk on peripheral artery disease risk: 0.755. Analyses were performed using fully adjusted Cox proportional hazards regression models, age, sex, ethnicity, Townsend deprivation index, education, body mass index, smoking status, alcohol intake frequency, sleep duration, physical activity, hypertension, dyslipidemia, diabetes, cardiovascular disease, aspirin use, genotype measurement batch, and first 10 genetic principal components.

In the genetic analyses, the constructed PRS for PAD demonstrated a significant positive association, yielding a HR of 1.18 (95% CI: 1.16–1.21) per SD increment (*P* for trend < 0.001; Supplementary Table 6). When analyses were stratified by genetic susceptibility, a higher frequency of adding salt to food remained associated with an increased PAD risk within each genetic risk stratum (Figure 2). No significant interaction was observed between the frequency of adding salt and genetic susceptibility on PAD risk (*P* for interaction = 0.755). Nevertheless, joint effect analysis demonstrated that individuals who always added salt and had high genetic risk faced a more than two-fold increased risk (HR = 2.16, 95% CI: 1.79–2.61) compared to the reference group with low-risk genetics and never/rarely added salt (Figure 2).

### 3.4 Stratified and sensitivity analyses

To assess potential effect modification, we performed stratified analyses by key covariates, including age, sex, BMI, lifestyle factors, and comorbidities (Supplementary Tables 7, 8). A significant interaction was observed between alcohol intake and salt-adding frequency for PAD risk (*P* for interaction = 0.029), with a more pronounced association among participants consuming alcohol ≥3 times per week. For CAS, the association with frequent salt addition was stronger in participants younger than 60 years (*P* for interaction = 0.036), and those without a history of CVD (*P* for interaction = 0.033). No significant interactions were detected for the other factors (all *P* for interaction > 0.05). Furthermore, the robustness of these findings was confirmed through a series of sensitivity analyses (Supplementary Tables 9, 10).

### 3.5 Metabolic signature for frequency of adding salt to foods and mediation analyses

**FIGURE 3:**
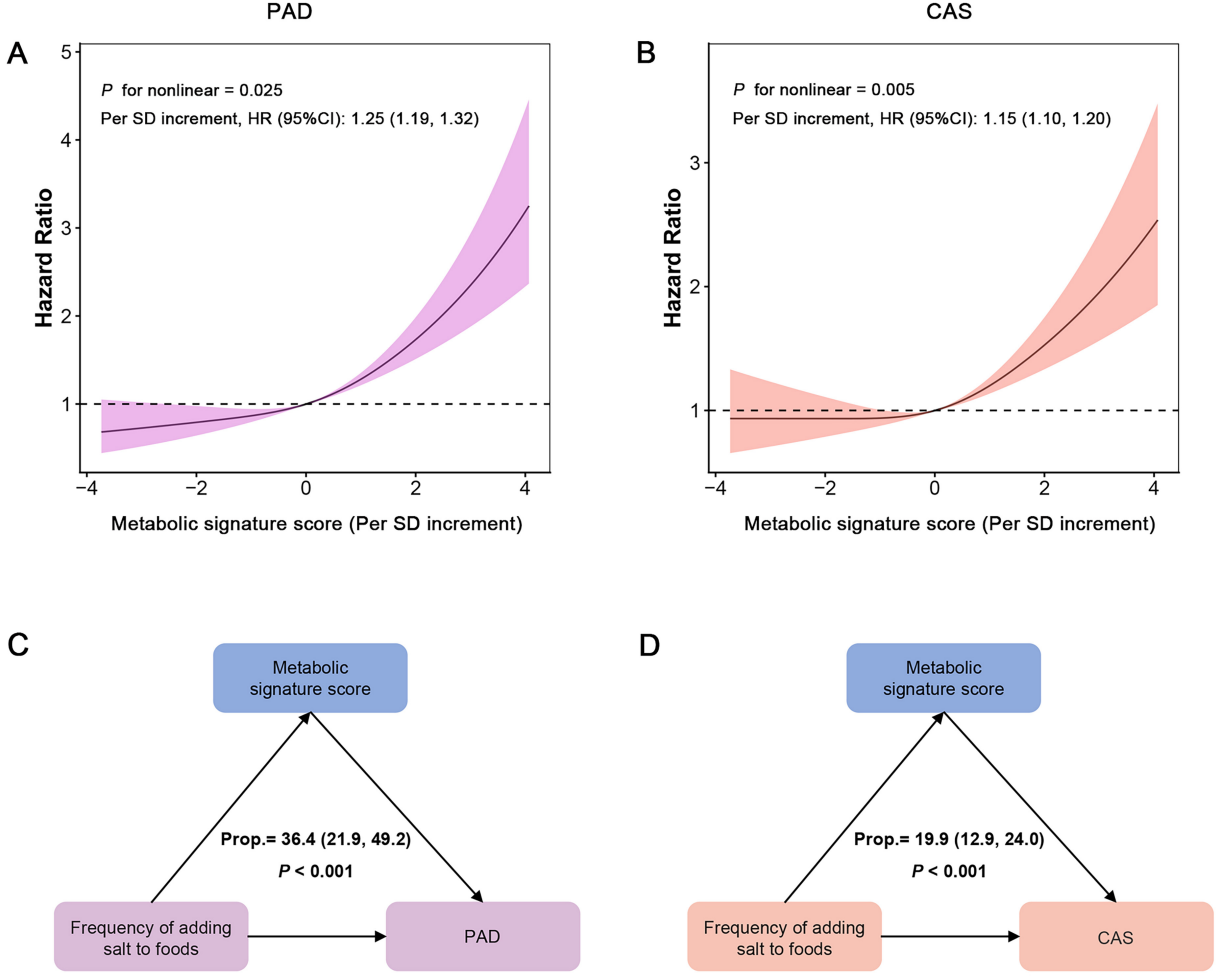
Metabolic signature mediates the link between frequency of adding salt to foods and extracoronary vascular disease. PAD, peripheral artery disease; CAS, carotid artery stenosis; SD, standard deviation; Prop., proportion. **(A,B)** Restricted cubic spline analyses assessing the potential nonlinear association between the metabolic signature score and risk of peripheral artery disease and carotid artery stenosis. **(C,D)** Mediation analyses evaluating the effect of the metabolic signature score on the association between the frequency of adding salt to foods and the risks of peripheral artery disease and carotid artery stenosis. Analyses were performed using fully adjusted Cox proportional hazards regression models, age, sex, ethnicity, Townsend deprivation index, education, body mass index, smoking status, alcohol intake frequency, sleep duration, physical activity, hypertension, dyslipidemia, diabetes, cardiovascular disease, and aspirin use.

We selected 26 out of 251 metabolites to construct the metabolic signature score for the frequency of adding salt to foods using LASSO regression. The selected metabolites included inflammatory markers, amino acids, fatty acids, ketone bodies, and lipoprotein lipids (Supplementary Table 11). Multivariate linear regression confirmed a positive association between frequent adding salt to foods (always vs. never/rarely) and the metabolic signature score (Coefficient = 0.3374, *P* < 0.001) (Supplementary Table 12). Among the specific metabolites, monounsaturated fatty acids to total fatty acids percentage showed the strongest positive association, whereas Omega-3 fatty acids and docosahexaenoic acid to total fatty acids percentage exhibited the strongest negative association (Supplementary Table 12). Per SD increment in this metabolic signature score was significantly associated with an increased risk of PAD (HR: 1.25, 95% CI: 1.19–1.32), and CAS (HR: 1.15, 95% CI: 1.10–1.20) (Figure 3). The relationships between the metabolic signature score and both PAD and CAS were nonlinear (both *P* for nonlinear < 0.05) (Figure 3). Additionally, of the 26 metabolites, we identified 19 individually associated with PAD or CAS risks. Glycoprotein acetyls showed the strongest positive association with both outcomes, whereas the linoleic acid to total fatty acids percentage was associated with a reduced risk. Mediation analysis indicated that this metabolic signature mediated 36.4% (95% CI: 21.9–49.2) and 19.9% (95% CI: 12.9–24.0) of the total effect of higher frequency of adding salt on PAD and CAS risks, respectively (Figure 3). Subsequent analysis of individual metabolites revealed 14 significant mediators, with estimated mediation proportions ranging from 0.8% to 11.7% (Supplementary Table 13).

## 4 Discussion

In this large prospective cohort study, we observed that a higher frequency of adding salt to foods was associated with an increased risk of extracoronary atherosclerotic vascular disease and related events. Genetic analyses further revealed that the association between salt-adding frequency and PAD was independent of genetic predisposition. We also developed a metabolic signature associated with salt-adding frequency. This metabolic profile statistically explained part of the observed association between salt-adding frequency and extracoronary atherosclerotic vascular disease risk.

### 4.1 Compared with previous studies

Previous studies have examined the relation between habitual salt use and cardiovascular health (11, 12). In particular, Liu et al. recently reported that people who added salt to foods more often had higher risks of microvascular, cerebrovascular, and CVD (12). Their study provided important population-based evidence that discretionary salt use may contribute to the burden of vascular disease. However, their work mainly focused on cardio-cerebrovascular diseases and did not specifically assess extracoronary atherosclerotic vascular outcomes, such as PAD, CAS, and related clinical events. This gap is worth addressing, as the pathogenesis of peripheral atherosclerosis may not be identical to that of coronary or cerebrovascular disease, particularly regarding the roles of hemodynamic stress (27), pathologic disparities (28), and metabolic dysregulation (29).

Beyond the choice of outcomes, previous research has predominantly employed cross-sectional designs and focused mainly on structural markers, such as plaque presence (9), carotid intima-media thickness (7), and arterial stiffness (8), without specifically tracking the incidence or progression of PAD or CAS. A recent Mendelian randomization study identified a relationship between salt added to food and the risk of PAD (IVW OR: 1.0040, 95% CI: 1.0015–1.0065) (30). However, the exposure variable in that study was imprecisely defined, failing to distinguish between salt added during cooking versus salt added at the table, potentially biasing sodium load estimation. In fact, conventional methods for assessing sodium intake, such as dietary questionnaires (31) or urinary sodium excretion measurements (32), have inherent limitations. Reliance on single-day measurements often introduces substantial random error, which can obscure the true direction of associations between sodium intake and health outcomes (33). In this study, we applied self-reported frequency of adding salt to foods as a practical behavioral indicator of discretionary salt intake in this study population. Previous work suggests this measure reflects long-term salt taste preference (11). It has also been shown to correlate positively with 24-h urinary sodium excretion (10), which supports its use as a reliable indicator.

In addition to validating the measure itself, we also sought to examine its biological correlates. Metabolomic analyses by Wuopio et al. have linked higher salt intake with multiple lipid species known to be associated with atherosclerosis (34), highlighting potential metabolic pathways through which salt intake may impact cardiovascular health. Notably, Tikkanen et al. found that the metabolic biomarker profiles associated with future risk differ between PAD and coronary artery disease, highlighting the pathophysiological heterogeneity across different vascular beds (29). In this context, our metabolomic analysis provides additional descriptive evidence that salt-adding behavior is associated with a broad circulating metabolic profile. Uniquely, our study demonstrates consistent links between Adding salt to foods, specific metabolic pathways, and the risk of two representative extracoronary vascular outcomes.

In the genetic susceptibility analyses, consistent with previous studies (14, 35), we observed no statistically significant interaction between the frequency of adding salt to foods and genetic predisposition to specific diseases. Our findings suggest that even individuals with a genetically lower risk of PAD may still benefit from limiting excessive salt consumption. In stratified analyses, the association was more pronounced among participants who consumed alcohol ≥3 times per week, suggesting a potential synergistic effect between high salt intake and regular alcohol consumption on PAD risk. Mechanistically, alcohol-mediated sympathetic activation and blood pressure variability may compound the hypertensive effect of high salt intake, leading to a greater overall hypertensive burden (36). Furthermore, both factors promote oxidative stress and inflammation, and their combined exposure may amplify endothelial injury and accelerate atherosclerosis (37, 38). Collectively, these findings suggest that frequent alcohol consumers may be particularly susceptible to the adverse vascular effects of habitual salt adding. The combination of salt reduction and alcohol moderation may therefore yield greater cardiovascular benefits than either strategy alone, especially for PAD prevention. Furthermore, the link between the frequency of adding salt to foods and CAS risk differed by age group and cardiovascular disease status. This suggests that salt reduction strategies for primary prevention should be tailored to these specific subpopulations.

### 4.2 Possible mechanisms

Elevated blood pressure has been widely recognized as the primary mediating pathway linking excessive salt intake to adverse cardiovascular outcomes (6, 9). Beyond blood pressure, our study identified a metabolic signature associated with frequency of adding salt to foods. This signature included metabolites related to inflammation, fatty acid, lipoprotein, ketone body, and amino acid. Several components of this profile may provide biological clues. Monounsaturated fatty acids to total fatty acids percentage have previously been implicated in increased PAD risk (29). Glycoprotein acetyls, a marker of inflammation, reflect both acute and low-grade chronic inflammatory states and may contribute to lipid metabolic disturbances (39) and endothelial dysfunction (40), thereby increasing the risk of extracoronary atherosclerotic vascular disease. Ketone bodies, intermediate products of hepatic fatty acid oxidation, have been shown to induce metabolic remodeling (40), oxidative stress (41), and inflammatory responses (42), all of which are closely associated with the development of extracoronary atherosclerotic vascular disease (43, 44). It is important to note that the biological effects of ketone bodies are highly context-dependent. Previous studies have demonstrated potential protective roles of β-hydroxybutyrate under various physiological and pathophysiological conditions. For instance, β-hydroxybutyrate has been shown to inhibit the NLRP3 inflammasome (45), act as a histone deacetylase inhibitor to modulate oxidative stress responses (46). Therefore, the associations observed in our study should not be interpreted as evidence that ketone bodies are universally pro-atherogenic. In addition, we observed inverse associations of omega-3 fatty acids to total fatty acids ratio, linoleic acid to total fatty acids ratio, docosahexaenoic acid to total fatty acids ratio, citrate, cholesteryl esters to total lipids in large low density lipoproteins ratio, and albumin with the disease. Further studies are warranted to validate these associations and to elucidate the underlying mechanisms linking these metabolites to the risk of extracoronary atherosclerotic vascular diseases.

### 4.3 Clinical implications

Peripheral artery disease and CAS often develop insidiously as subclinical manifestations of atherosclerotic vascular disease, yet their clinical consequences can be severe. Current primary prevention strategies primarily target traditional risk factors, aiming to mitigate inflammation and thrombosis. However, a survey conducted in England revealed that over 40% of participants habitually add salt to their food (47), highlighting an underrecognized and modifiable lifestyle risk factor that has been largely overlooked in existing prevention efforts. Our findings advance the understanding of the systemic vascular effects of high salt intake and identify discretionary salt use as a cost-effective and readily implementable target for primary prevention. Importantly, long-term follow-up of sodium reduction interventions in the Trials of Hypertension Prevention showed that reducing dietary sodium intake was associated with a lower risk of cardiovascular events among adults with prehypertension (48). This robust clinical evidence supports the feasibility and clinical relevance of interventions focused on reducing discretionary salt consumption, underscoring the translational potential of our results.

### 4.4 Strengths and limitations

Our study has several strengths, including a large sample size, extended follow-up duration, consistent results across multiple sensitivity analyses, and the integration of metabolomics to identify circulating metabolites associated with salt-adding frequency and extracoronary atherosclerotic vascular disease risk. Nevertheless, several limitations should be acknowledged. First, the exposure variable, self-reported frequency of adding salt to food, does not provide quantitative estimates of total sodium intake. However, previous studies have demonstrated a dose-response relationship between this variable and urinary sodium concentration (10), supporting its use as a practical indicator of habitual salt exposure and has been validated in multiple cohorts (14, 23, 49). Second, the frequency of adding salt to foods was assessed only once at baseline, and individual preferences may change over time. However, Nevertheless, the frequency of adding salt to foods remained largely stable over 4 years, with a weighted κ of 0.71 between baseline and the first reassessment using touchscreen questionnaires (50). We also conducted a sensitivity analysis excluding participants who reported major dietary changes in the past 5 years, and the results remained consistent. Third, although the prospective longitudinal design establishes the temporal sequence, causality cannot be established. Despite adjusting for a wide range of covariates, residual confounding cannot be fully excluded, as lifestyle and dietary behaviors are complex and imperfectly measured. In particular, unmeasured dietary factors, such as higher intake of processed or salty foods, may have partly accounted for the observed associations. Nonetheless, the consistent positive associations across sensitivity analyses and the significant dose-response trend lend some support to the robustness of the findings. Finally, the study population consisted predominantly of individuals of European ancestry, which may limit the generalizability of our findings to other racial or ethnic groups.

## 5 Conclusion

In conclusion, frequent adding of salt to foods was associated with higher risks of extracoronary atherosclerotic vascular disease and related clinical events. A salt-adding behavior-associated metabolic profile statistically explained part of these associations.

## Data Availability

The data analyzed in this study is subject to the following licenses/restrictions: The data that support the findings of this study are available from UK Biobank. These data are subject to access restrictions and cannot be shared by the authors. However, the UK Biobank resource is available to all eligible researchers upon application and approval. Requests to access these datasets should be directed to UK Biobank (www.ukbiobank.ac.uk).

## References

[1] Mazzolai L Teixido-Tura G Lanzi S Boc V Bossone E Brodmann M et al. 2024 ESC Guidelines for the management of peripheral arterial and aortic diseases Developed by the task force on the management of peripheral arterial and aortic diseases of the European Society of Cardiology (ESC) Endorsed by the European Association for Cardio-Thoracic Surgery (EACTS), the European Reference Network on Rare Multisystemic Vascular Diseases (VASCERN), and the European Society of Vascular Medicine (ESVM). *Eur Heart J.* (2024) 45:3538–700. 10.1093/eurheartj/ehae179 39210722

[2] Criqui M Aboyans V. Epidemiology of peripheral artery disease. *Circ Res.* (2015) 116:1509–26. 10.1161/CIRCRESAHA.116.303849 25908725

[3] de Weerd M Greving J de Jong A Buskens E Bots M. Prevalence of asymptomatic carotid artery stenosis according to age and sex: systematic review and metaregression analysis. *Stroke.* (2009) 40:1105–13. 10.1161/STROKEAHA.108.532218 19246704

[4] Saratzis A Jaspers N Gwilym B Thomas O Tsui A Lefroy R et al. Observational study of the medical management of patients with peripheral artery disease. *Br J Surg.* (2019) 106:1168–77. 10.1002/bjs.11214 31259387

[5] McDermott M Mandapat A Moates A Albay M Chiou E Celic L et al. Knowledge and attitudes regarding cardiovascular disease risk and prevention in patients with coronary or peripheral arterial disease. *Arch Intern Med.* (2003) 163:2157–62. 10.1001/archinte.163.18.2157 14557213

[6] O’Donnell M Mente A Yusuf S. Sodium intake and cardiovascular health. *Circ Res.* (2015) 116:1046–57. 10.1161/CIRCRESAHA.116.303771 25767289

[7] Peng S Wang J Xiao Y Yin L Peng Y Yang L et al. The association of carotid artery atherosclerosis with the estimated excretion levels of urinary sodium and potassium and their ratio in Chinese adults. *Nutr J.* (2021) 20:50. 10.1186/s12937-021-00710-8 34092243 PMC8182948

[8] Tsirimiagkou C Karatzi K Argyris A Chalkidou F Tzelefa V Sfikakis P et al. Levels of dietary sodium intake: diverging associations with arterial stiffness and atheromatosis. *Hellenic J Cardiol.* (2021) 62:439–46. 10.1016/j.hjc.2021.02.005 33610752

[9] Wuopio J Ling Y Orho-Melander M Engström G Ärnlöv J. The association between sodium intake and coronary and carotid atherosclerosis in the general Swedish population. *Eur Heart J Open.* (2023) 3:oead024. 10.1093/ehjopen/oead024 37006408 PMC10063371

[10] Ma H Xue Q Wang X Li X Franco O Li Y et al. Adding salt to foods and hazard of premature mortality. *Eur Heart J.* (2022) 43:2878–88. 10.1093/eurheartj/ehac208 35808995 PMC9890626

[11] Ma H Wang X Li X Heianza Y Qi L. Adding salt to foods and risk of cardiovascular disease. *J Am Coll Cardiol.* (2022) 80:2157–67. 10.1016/j.jacc.2022.09.039 36456045

[12] Liu M Ye Z He P Yang S Zhang Y Zhou C et al. Adding salt to foods and hazards of microvascular, cerebrovascular and cardiovascular diseases. *Eur J Clin Nutr.* (2024) 78:141–8. 10.1038/s41430-023-01354-z 37838806

[13] Park Y Yang P Park B Park J Jang E Kim D et al. Association of adding salt to foods and potassium intake with incident atrial fibrillation in the UK Biobank Study. *Rev Cardiovasc Med.* (2024) 25:332. 10.31083/j.rcm2509332 39355602 PMC11440396

[14] Zhao Y Li Y Zhuang Z Song Z Jia J Huang T. Frequency of adding salt to foods, genetic susceptibility, and incident type 2 diabetes: a prospective cohort study. *J Clin Endocrinol Metab.* (2024) 109:e589–95. 10.1210/clinem/dgad544 37758206

[15] Smith E Ericson U Hellstrand S Orho-Melander M Nilsson P Fernandez C et al. A healthy dietary metabolic signature is associated with a lower risk for type 2 diabetes and coronary artery disease. *BMC Med.* (2022) 20:122. 10.1186/s12916-022-02326-z 35443726 PMC9022292

[16] Wei C Gong W Xu B Yu B Zhou S Zhu Z. Associations between sweetened beverage consumption, degenerative valvular heart disease, and related events: a prospective study from UK Biobank. *Eur J Prev Cardiol.* (2025) 32:1763–75. 10.1093/eurjpc/zwaf293 40359385

[17] Sudlow C Gallacher J Allen N Beral V Burton P Danesh J et al. UK biobank: an open access resource for identifying the causes of a wide range of complex diseases of middle and old age. *PLoS Med.* (2015) 12:e1001779. 10.1371/journal.pmed.1001779 25826379 PMC4380465

[18] Bellomo T Bramel E Lee J Urbut S Flores A Yu Z et al. Evaluation of Lipoprotein(a) as a prognostic marker of extracoronary atherosclerotic vascular disease progression. *Circulation.* (2025) 152:585–98. 10.1161/CIRCULATIONAHA.124.073579 40718930 PMC12313207

[19] Flores A Ruan Y Misra A Cho S Selvaraj M Bellomo T et al. Polygenic prediction of peripheral artery disease and major adverse limb events. *JAMA Cardiol.* (2025) 10:770–8. 10.1001/jamacardio.2025.1182 40397457 PMC12096330

[20] Zhu K Qian F Lu Q Li R Qiu Z Li L et al. Modifiable lifestyle factors, genetic risk, and incident peripheral artery disease among individuals with type 2 diabetes: a prospective study. *Diabetes Care.* (2024) 47:435–43. 10.2337/dc23-1503 38181303

[21] Bull F Al-Ansari S Biddle S Borodulin K Buman M Cardon G et al. World Health Organization 2020 guidelines on physical activity and sedentary behaviour. *Br J Sports Med.* (2020) 54:1451–62. 10.1136/bjsports-2020-102955 33239350 PMC7719906

[22] Inker L Eneanya N Coresh J Tighiouart H Wang D Sang Y et al. New creatinine- and cystatin c-based equations to estimate GFR without race. *N Engl J Med.* (2021) 385:1737–49. 10.1056/NEJMoa2102953 34554658 PMC8822996

[23] Tang R Kou M Wang X Ma H Li X Heianza Y et al. Self-reported frequency of adding salt to food and risk of incident chronic kidney disease. *JAMA Netw Open.* (2023) 6:e2349930. 10.1001/jamanetworkopen.2023.49930 38153731 PMC10755616

[24] Klarin D Lynch J Aragam K Chaffin M Assimes T Huang J et al. Genome-wide association study of peripheral artery disease in the million veteran program. *Nat Med.* (2019) 25:1274–9. 10.1038/s41591-019-0492-5 31285632 PMC6768096

[25] Cao C Wei C Gong W Yu B Fang Z Zhou S et al. Social disconnection, genetic risk, and the incidence of degenerative valvular heart disease: a population-based cohort study. *J Am Heart Assoc.* (2026) 15:e045931. 10.1161/JAHA.125.045931 41983291 PMC13279164

[26] Julkunen H Cichońska A Tiainen M Koskela H Nybo K Mäkelä V et al. Atlas of plasma NMR biomarkers for health and disease in 118,461 individuals from the UK Biobank. *Nat Commun.* (2023) 14:604. 10.1038/s41467-023-36231-7 36737450 PMC9898515

[27] Cecchi E Giglioli C Valente S Lazzeri C Gensini G Abbate R et al. Role of hemodynamic shear stress in cardiovascular disease. *Atherosclerosis.* (2011) 214:249–56. 10.1016/j.atherosclerosis.2010.09.008 20970139

[28] Narula N Olin J Narula N. Pathologic disparities between peripheral artery disease and coronary artery disease. *Arterioscler Thromb Vasc Biol.* (2020) 40:1982–9. 10.1161/ATVBAHA.119.312864 32673526

[29] Tikkanen E Jägerroos V Holmes M Sattar N Ala-Korpela M Jousilahti P et al. Metabolic biomarker discovery for risk of peripheral artery disease compared with coronary artery disease: lipoprotein and metabolite profiling of 31 657 individuals from 5 prospective cohorts. *J Am Heart Assoc.* (2021) 10:e021995. 10.1161/JAHA.121.021995 34845932 PMC9075369

[30] Wang S Wang Y Lu X Wang H Sun J Wang X. Association of salt added to food with risk of cardiovascular diseases: a 2-sample Mendelian randomization study. *Medicine.* (2025) 104:e41543. 10.1097/MD.0000000000041543 40101059 PMC11922462

[31] Cogswell M Mugavero K Bowman B Frieden T. Dietary sodium and cardiovascular disease risk–measurement matters. *N Engl J Med.* (2016) 375:580–6. 10.1056/NEJMsb1607161 27248297 PMC5381724

[32] Dougher C Rifkin D Anderson C Smits G Persky M Block G et al. Spot urine sodium measurements do not accurately estimate dietary sodium intake in chronic kidney disease. *Am J Clin Nutr.* (2016) 104:298–305. 10.3945/ajcn.115.127423 27357090 PMC4962156

[33] Cobb L Anderson C Elliott P Hu F Liu K Neaton J et al. Methodological issues in cohort studies that relate sodium intake to cardiovascular disease outcomes: a science advisory from the American heart association. *Circulation.* (2014) 129:1173–86. 10.1161/CIR.0000000000000015 24515991

[34] Wuopio J Yi-Ting L Dekkers K Fall T Smith J Larsson A et al. The metabolic signature of salt intake: a cross-sectional analysis from the SCAPIS-study. *Nutr Metab.* (2025) 22:104. 10.1186/s12986-025-00997-y 40898250 PMC12406461

[35] Chen H Zhang X Lin S Wu Q. Adding salt to foods increases the risk of metabolic dysfunction-associated steatotic liver disease. *Commun Med.* (2025) 5:342. 10.1038/s43856-025-01074-4 40781351 PMC12334587

[36] Nan X Lu H Wu J Xue M Qian Y Wang W et al. The interactive association between sodium intake, alcohol consumption and hypertension among elderly in northern China: a cross-sectional study. *BMC Geriatr.* (2021) 21:135. 10.1186/s12877-021-02090-4 33622268 PMC7903677

[37] Surma S Okopień B Murphy A Banach M. High salt intake and atherosclerosis progression-not only via blood pressure: a narrative review. *Nutrients.* (2025) 17:3464. 10.3390/nu17213464 41228536 PMC12610747

[38] Piano M Marcus G Aycock D Buckman J Hwang C Larsson S et al. Alcohol use and cardiovascular disease: a scientific statement from the American heart association. *Circulation.* (2025) 152:e7–21. 10.1161/CIR.0000000000001341 40485439

[39] Esteve E Ricart W Fernández-Real J. Dyslipidemia and inflammation: an evolutionary conserved mechanism. *Clin Nutr.* (2005) 24:16–31. 10.1016/j.clnu.2004.08.004 15681098

[40] Jain V Chevli P Garg P McParland J Kizer J Mukamal K et al. Circulating ketone bodies and risk of incident atrial fibrillation: insights from the MESA and UK Biobank cohorts. *Eur J Prev Cardiol.* (2025): 10.1093/eurjpc/zwaf543 Online ahead of print. 40991362

[41] Jain S McVie R. Hyperketonemia can increase lipid peroxidation and lower glutathione levels in human erythrocytes in vitro and in type 1 diabetic patients. *Diabetes.* (1999) 48:1850–5. 10.2337/diabetes.48.9.1850 10480618

[42] Shi X Li X Li D Li Y Song Y Deng Q et al. β-Hydroxybutyrate activates the NF-κB signaling pathway to promote the expression of pro-inflammatory factors in calf hepatocytes. *Cell Physiol Biochem.* (2014) 33:920–32. 10.1159/000358664 24713665

[43] Aboyans V Canonico M Chastaingt L Anand S Brodmann M Couffinhal T et al. Peripheral artery disease. *Nat Rev Dis Primers.* (2025) 11:68. 10.1038/s41572-025-00651-0 40968271

[44] Klarin D Tsao P Damrauer S. Genetic determinants of peripheral artery disease. *Circ Res.* (2021) 128:1805–17. 10.1161/CIRCRESAHA.121.318327 34110906

[45] Youm Y Nguyen K Grant R Goldberg E Bodogai M Kim D et al. The ketone metabolite β-hydroxybutyrate blocks NLRP3 inflammasome-mediated inflammatory disease. *Nat Med.* (2015) 21:263–9. 10.1038/nm.3804 25686106 PMC4352123

[46] Shimazu T Hirschey M Newman J He W Shirakawa K Le Moan N et al. Suppression of oxidative stress by β-hydroxybutyrate, an endogenous histone deacetylase inhibitor. *Science.* (2013) 339:211–4. 10.1126/science.1227166 23223453 PMC3735349

[47] Millett C Laverty A Stylianou N Bibbins-Domingo K Pape U. Impacts of a national strategy to reduce population salt intake in England: serial cross sectional study. *PLoS One.* (2012) 7:e29836. 10.1371/journal.pone.0029836 22238665 PMC3251604

[48] Cook N Cutler J Obarzanek E Buring J Rexrode K Kumanyika S et al. Long term effects of dietary sodium reduction on cardiovascular disease outcomes: observational follow-up of the trials of hypertension prevention (TOHP). *BMJ.* (2007) 334:885–8. 10.1136/bmj.39147.604896.55 17449506 PMC1857760

[49] Zhou G Gan L Zhao B Fang F Liu H Chen X et al. Adding salt to foods and risk of psoriasis: a prospective cohort study. *J Autoimmun.* (2024) 147:103259. 10.1016/j.jaut.2024.103259 38823158

[50] Bradbury K Young H Guo W Key T. Dietary assessment in UK Biobank: an evaluation of the performance of the touchscreen dietary questionnaire. *J Nutr Sci.* (2018) 7:e6. 10.1017/jns.2017.66 29430297 PMC5799609

