# Supplementary material for: Frequency of adding salt to foods, metabolic signature, and the risk of extracoronary atherosclerotic vascular disease progression
Source: Front Nutr. 2026 Jul 24;13:1870553. doi: 10.3389/fnut.2026.1870553 (PMC13447142; doi:10.3389/fnut.2026.1870553)
Supplement: Supplementary file 1 [file Table_1.docx]

Supplementary Material

# 1 Supplementary Figures and Tables

## 1.1 Supplementary Figures

**Supplementary Figure 1.** **The flowchart about the inclusion and exclusion of study participants.**

## 1.3 Supplementary Tables

**Supplementary Table 1 Definition of outcomes in the UK Biobank.**

| **Disease** | **Data field ID** | **ICD-10/OPCS-4** |
| --- | --- | --- |
| **Peripheral artery disease** |  |  |
| 1. Underlying (primary/ secondary) cause of death ICD-10; Diagnoses - ICD-10 | 40001; 40002; 41270; 41280 | I70.0; I70.00; I70.01; I70.2; I70.20; I70.21; I70.8; I70.80; I70.9; I70.90; I73.8; I73.9 |
| 2. Operative procedures - OPCS4 | 41272; 41282 | X09.3; X09.4; X09.5; L21.6; L51.3; L51.6; L51.8; L52.1; L52.2; L54.1; L54.4; L54.8; L59.1; L59.2; L59.3; L59.4; L59.5; L59.6; L59.7; L59.8; L60.1; L60.2; L63.1; L63.5; L63.9; L66.7 |
| **Major adverse limb events** |  |  |
| 1. Underlying (primary/ secondary) cause of death ICD-10; Diagnoses - ICD-10 | 40001; 40002; 41270; 41280 | I74.0; I74.3; I74.4; I74.5 |
| 2. Operative procedures - OPCS4 | 41272; 41282 | X09.1; X09.2; X09.3; X09.4; 09.5; X09.8; X09.9; X10.1; X10.8; 10.9; X12.1; X12.3; L16.1; L25.3; L26.3; L50.3; L50.4; L50.6; L50.8; L50.9; L53.2; L54.2; L58.1; L58.2; L58.3; L58.4; L58.5; L58.6; L58.7; L58.8; L58.9; L62.2; L63.2; L66.1; L70.1; L71.2; L71.6 |
| **Carotid artery stenosis** |  |  |
| 1. Underlying (primary/ secondary) cause of death ICD-10; Diagnoses - ICD-10 | 40001; 40002; 41270;41280 | G45.9; I63.0; I63.1; I63.2; I65.2; I65.3; I65.8; I65.9; 67.2; I69.4 |
| 2. Operative procedures - OPCS4 | 41272;41282 | L29.4; L29.5; L31.1; L31.2; L31.3; L31.4; L31.8; L31.9 |
| **Stroke** |  |  |
| 1. Underlying (primary/ secondary) cause of death ICD-10; Diagnoses - ICD-10 | 40001; 40002; 41270; 41280 | I63.0; I63.1; I63.2; I63.3; I63.4; I63.5; I63.8; I63.9; I64.0; I69.3; I69.4 |

Abbreviations: ICD: international classification disease; OPCS: office of population censuses and surveys classification of interventions and procedures.

All outcomes were ascertained from three separate sources: non-fatal events from hospital records, fatal events from death registries, and procedural records.

**Supplementary Table 2 Components and criteria for diet score.**

| **Diet components** | **Field ID** | **Healthy diet score** |
| --- | --- | --- |
| Fruits | 1309 (fresh fruit/day intake)  1319 (dried fruit/day intake) | 1 for ≥ 3 pieces/day  0 for < 3 pieces/day |
| Vegetables | 1289 (cooked vegetables/day intake)  1299 (salad/raw vegetables/day intake) | 1 for ≥ 4 tablespoons/day  0 for < 4 tablespoons/day |
| Fish | 1329 (oily fish intake)  1399 (non-oily fish intake) | 1 for ≥ 2 times/week  0 for < 2 times/week |
| Unprocessed meat | 1369 (beef intake)  1379 (lamb/mutton intake)  1389 (pork intake) | 1 for < 2 times/week  0 for ≥ 2 times/week |
| Processed meat | 1349 (processed meat intake) | 1 for < 2 times/week  0 for ≥ 2 times/week |

The sum of five diet components is the overall diet score, range from 0-5. A higher score indicates a better diet score.

**Supplementary Table 3 The numbers and percentages of participants with missing covariates.**

| **Covariates** | **Missing numbers** | **Missing percentages (%)** |
| --- | --- | --- |
| Ethnicity | 1,592 | 0.3 |
| TDI | 609 | 0.1 |
| Education | 8,954 | 1.8 |
| BMI | 2,361 | 0.5 |
| Alcohol intake frequency | 368 | <0.1 |
| Smoking status | 1,647 | 0.3 |
| Physical activity | 105,580 | 21.3 |
| Sleep duration | 708 | 0.1 |
| Diet score | 7,014 | 1.4 |

Abbreviations: TDI, Townsend deprivation index; BMI, body mass index

**Supplementary Table 4 Summary of genetic variants for polygenic risk scores construction.**

| **SNP** | **Chromosome** | **Risk/reference allele** | **OR (95% CI)** | ***P*** |
| --- | --- | --- | --- | --- |
| rs7528419 | 1 | A/G | 1.07 (1.05, 1.09) | 2.54 × 10^–11^ |
| rs6025 | 1 | T/C | 1.20 (1.14, 1.26) | 1.63 × 10^–12^ |
| rs118039278 | 6 | A/G | 1.26 (1.22, 1.30) | 1.57 × 10^–43^ |
| rs3130968 | 6 | T/C | 1.07 (1.05, 1.10) | 3.16 × 10^–10^ |
| rs2107595 | 7 | A/G | 1.08 (1.05, 1.10) | 2.49 × 10^–11^ |
| rs4722172 | 7 | G/A | 1.08 (1.05, 1.10) | 3.65 × 10^–11^ |
| rs322 | 8 | A/C | 1.06 (1.04, 1.07) | 2.53 × 10^–9^ |
| rs505922 | 9 | C/T | 1.06 (1.04, 1.07) | 7.10 × 10^–11^ |
| rs1537372 | 9 | T/G | 1.12 (1.10, 1.14) | 4.32 × 10^–39^ |
| rs7903146 | 10 | T/C | 1.06 (1.04, 1.08) | 3.76 × 10^–11^ |
| rs566125 | 11 | T/C | 1.08 (1.05, 1.11) | 4.37 × 10^–9^ |
| rs7476 | 11 | C/A | 1.06 (1.04, 1.08) | 8.33 × 10^–10^ |
| rs11066301 | 12 | G/A | 1.06 (1.04, 1.08) | 2.96 × 10^–11^ |
| rs4842266 | 12 | G/A | 1.06 (1.04, 1.08) | 1.01 × 10^–9^ |
| rs1975514 | 13 | C/T | 1.05 (1.04, 1.07) | 8.32 × 10^–10^ |
| rs55784307 | 14 | A/C | 1.06 (1.04, 1.09) | 2.93 × 10^–8^ |
| rs10851907 | 15 | A/G | 1.06 (1.05, 1.08) | 1.49 × 10^–13^ |
| rs62084752 | 17 | C/G | 1.07 (1.05, 1.09) | 1.58 × 10^–10^ |
| rs138294113 | 19 | C/T | 1.09 (1.06, 1.11) | 1.20 × 10^–10^ |

Abbreviations: SNP, single nucleotide polymorphism; OR, odds ratio.

**Supplementary Table 5 Baseline characteristics of participants with or without peripheral artery disease and carotid artery stenosis.**

| **Characteristic** | **PAD** | |  | **CAS** | |
| --- | --- | --- | --- | --- | --- |
|  | **No** | **Yes** |  | **No** | **Yes** |
| **Participants, n** | 487,830 | 7,461 |  | 487,224 | 8,067 |
| **Age in years, median [IQR]** | 58.0 (50.0, 63.0) | 63.0 (59.0, 66.0) |  | 58.0 (50.0, 63.0) | 63.0 (58.0, 66.0) |
| **Sex, n (%)** |  |  |  |  |  |
| Female | 267,721 (55%) | 2,692 (36%) |  | 267,018 (55%) | 3,395 (42%) |
| Male | 220,109 (45%) | 4,769 (64%) |  | 220,206 (45%) | 4,672 (58%) |
| **White ethnicity, n (%)** | 459,783 (94%) | 7,120 (95%) |  | 459,264 (94%) | 7,639 (95%) |
| **TDI, median [IQR]** | -2.2 (-3.7, 0.5) | -1.3 (-3.2, 2.1) |  | -2.2 (-3.7, 0.5) | -1.8 (-3.4, 1.4) |
| **eGFR, median [IQR] ml/min/1.73 m^2^** | 96.1 (86.6, 105.2) | 88.8 (77.8, 98.4) |  | 96.1 (86.6, 105.2) | 89.7 (79.4, 99.3) |
| **BMI, n (%)** |  |  |  |  |  |
| < 30 kg/m2 | 370,336 (76%) | 4,878 (65%) |  | 369,683 (76%) | 5,531 (69%) |
| ≥ 30 kg/m2 | 117,494 (24%) | 2,583 (35%) |  | 117,541 (24%) | 2,536 (31%) |
| **Education, n (%)** |  |  |  |  |  |
| College or university | 158,297 (32%) | 1,321 (18%) |  | 157,797 (32%) | 1,821 (23%) |
| Others | 320,782 (66%) | 5,928 (79%) |  | 320,673 (66%) | 6,037 (75%) |
| Unknown | 8,751 (1.8%) | 212 (2.8%) |  | 8,754 (1.8%) | 209 (2.6%) |
| **Smoking status, n (%)** |  |  |  |  |  |
| Never | 269,256 (55%) | 2,125 (28%) |  | 267,844 (55%) | 3,537 (44%) |
| Previous | 166,995 (34%) | 3,264 (44%) |  | 166,981 (34%) | 3,278 (41%) |
| Current | 49,755 (10%) | 2,025 (27%) |  | 50,580 (10%) | 1,200 (15%) |
| Unknown | 1,824 (0.4%) | 47 (0.6%) |  | 1,819 (0.4%) | 52 (0.6%) |
| **Alcohol intake frequency, n (%)** |  |  |  |  |  |
| < 3 times/week | 275,775 (57%) | 4,296 (58%) |  | 275,370 (57%) | 4,701 (58%) |
| ≥ 3 times/week | 211,629 (43%) | 3,147 (42%) |  | 211,425 (43%) | 3,351 (42%) |
| Unknown | 426 (<0.1%) | 18 (0.2%) |  | 429 (<0.1%) | 15 (0.2%) |
| **Physical activity, n (%)** |  |  |  |  |  |
| < 150 min/week | 71,309 (15%) | 1,352 (18%) |  | 71,293 (15%) | 1,368 (17%) |
| ≥ 150 min/week | 304,236 (62%) | 3,878 (52%) |  | 303,619 (62%) | 4,495 (56%) |
| Unknown | 112,285 (23%) | 2,231 (30%) |  | 112,312 (23%) | 2,204 (27%) |
| **Sleep duration, n (%)** |  |  |  |  |  |
| Normal, 7-8 h/d | 328,706 (67%) | 4,335 (58%) |  | 328,136 (67%) | 4,905 (61%) |
| Short, < 7 h/d | 119,404 (24%) | 2,143 (29%) |  | 119,406 (25%) | 2,141 (27%) |
| Long, > 8 h/d | 36,706 (7.5%) | 891 (12%) |  | 36,664 (7.5%) | 933 (12%) |
| Unknown | 3,014 (0.6%) | 92 (1.2%) |  | 3,018 (0.6%) | 88 (1.1%) |
| **Diet score, n (%)** |  |  |  |  |  |
| < 3 | 235,348 (48%) | 3,590 (48%) |  | 235,149 (48%) | 3,789 (47%) |
| ≥ 3 | 252,482 (52%) | 3,871 (52%) |  | 252,075 (52%) | 4,278 (53%) |
| **Comorbidities, n (%)** |  |  |  |  |  |
| Dyslipidemia | 70,537 (14%) | 2,611 (35%) |  | 70,741 (15%) | 2,407 (30%) |
| Diabetes | 12,369 (2.5%) | 841 (11%) |  | 12,586 (2.6%) | 624 (7.7%) |
| Hypertension | 127,619 (26%) | 3,941 (53%) |  | 127,622 (26%) | 3,938 (49%) |
| CVD | 23,421 (4.8%) | 1,556 (21%) |  | 23,129 (4.7%) | 1,848 (23%) |
| AF | 7,608 (1.6%) | 410 (5.5%) |  | 7,626 (1.6%) | 392 (4.9%) |
| CKD | 5,486 (1.1%) | 278 (3.7%) |  | 5,553 (1.1%) | 211 (2.6%) |
| **Medication, n (%)** |  |  |  |  |  |
| Aspirin | 64,150 (13%) | 2,688 (36%) |  | 64,312 (13%) | 2,526 (31%) |
| Lipid-lowering medication | 80,270 (16%) | 3,363 (45%) |  | 80,553 (17%) | 3,080 (38%) |
| Diabetes medication | 17,094 (3.5%) | 1,204 (16%) |  | 17,462 (3.6%) | 836 (10%) |
| Antihypertensive medication | 97,595 (20%) | 3,511 (47%) |  | 97,734 (20%) | 3,372 (42%) |

Abbreviations: PAD, Peripheral artery disease; CAS, carotid artery stenosis; IQR, interquartile range; TDI, Townsend deprivation index; BMI, body mass index; CVD, cardiovascular disease; AF, atrial fibrillation; CKD, chronic kidney disease.

Categorical variables are presented as numbers (percentages). Continuous variables are presented as median (interquartile range).

**Supplementary Table 6 Risk of incident peripheral artery disease at different genetic risk stratified by polygenic risk score.**

| **PRS** | **Cases/N** | **Model 1** |  | **Model 2** |  |
| --- | --- | --- | --- | --- | --- |
|  |  | **HR (95% CI)** | ***P*** | **HR (95% CI)** | ***P*** |
| **Per SD increment** | 9,162/458,754 | 1.22 (1.20-1.25) | <0.001 | 1.18 (1.16-1.21) | <0.001 |
| **PRS category** |  |  |  |  |  |
| Low | 1,401/91,753 | 1.00 (reference) |  | 1.00 (reference) |  |
| Intermediate | 5,316/275,250 | 1.27 (1.20-1.35) | <0.001 | 1.23 (1.16-1.30) | <0.001 |
| High | 2,445/91,751 | 1.78 (1.66-1.90) | <0.001 | 1.62 (1.51-1.73) | <0.001 |
| *P* for trend |  | <0.001 | | <0.001 | |

Abbreviations: PRS, polygenic risk score; SD, standard deviation.

Analyses were performed using fully adjusted Cox proportional hazards regression models, age, sex, ethnicity, Townsend deprivation index, education, body mass index, smoking status, alcohol intake frequency, sleep duration, physical activity, hypertension, dyslipidemia, diabetes, cardiovascular disease, aspirin use, genotype measurement batch, and first 10 genetic principal components.

**Supplementary Table 7 Stratified analyses for the association between frequency of adding salt to foods and the incidence of peripheral artery disease.**

| **Subgroup** | **Frequency of adding salt to foods, HR (95% CI)** | | | | ***P* for interaction** |
| --- | --- | --- | --- | --- | --- |
|  | **Never/rarely** | **Sometimes** | **Usually** | **Always** |  |
| **Age** |  |  |  |  | 0.145 |
| < 60 years | 1.00 (reference) | 1.09 (0.98-1.20) | 1.25 (1.10-1.42) | 1.41 (1.21-1.65) |  |
| ≥ 60 years | 1.00 (reference) | 1.08 (1.01-1.15) | 1.13 (1.04-1.22) | 1.32 (1.18-1.46) |  |
| **Sex** |  |  |  |  | 0.798 |
| Female | 1.00 (reference) | 1.05 (0.96-1.14) | 1.12 (0.99-1.26) | 1.28 (1.10-1.49) |  |
| Male | 1.00 (reference) | 1.10 (1.03-1.18) | 1.19 (1.09-1.29) | 1.40 (1.25-1.55) |  |
| **BMI, kg/m^2^** |  |  |  |  | 0.416 |
| < 30 kg/m^2^ | 1.00 (reference) | 1.10 (1.03-1.17) | 1.15 (1.06-1.25) | 1.33 (1.19-1.48) |  |
| ≥ 30 kg/m^2^ | 1.00 (reference) | 1.03 (0.94-1.13) | 1.15 (1.02-1.28) | 1.35 (1.17-1.57) |  |
| **Smoking status** |  |  |  |  | 0.382 |
| Non-current | 1.00 (reference) | 1.07 (1.01-1.14) | 1.20 (1.10-1.30) | 1.32 (1.17-1.48) |  |
| Current | 1.00 (reference) | 1.09 (0.98-1.21) | 1.07 (0.94-1.21) | 1.34 (1.17-1.54) |  |
| **Alcohol intake frequency** |  |  |  |  | **0.029** |
| < 3 times/week | 1.00 (reference) | 1.09 (1.02-1.17) | 1.14 (1.04-1.25) | 1.23 (1.09-1.38) |  |
| ≥ 3 times/week | 1.00 (reference) | 1.07 (0.99-1.17) | 1.20 (1.08-1.32) | 1.53 (1.34-1.74) |  |
| **Dyslipidemia** |  |  |  |  | 0.562 |
| No | 1.00 (reference) | 1.07 (1.00-1.15) | 1.17 (1.08-1.27) | 1.38 (1.24-1.53) |  |
| Yes | 1.00 (reference) | 1.11 (1.01-1.21) | 1.16 (1.03-1.30) | 1.31 (1.12-1.53) |  |
| **Diabetes** |  |  |  |  | 0.098 |
| No | 1.00 (reference) | 1.09 (1.03-1.15) | 1.17 (1.09-1.26) | 1.32 (1.20-1.45) |  |
| Yes | 1.00 (reference) | 1.02 (0.87-1.19) | 1.00 (0.81-1.24) | 1.59 (1.23-2.05) |  |
| **CVD** |  |  |  |  | 0.206 |
| No | 1.00 (reference) | 1.06 (1.00-1.13) | 1.16 (1.08-1.26) | 1.36 (1.23-1.50) |  |
| Yes | 1.00 (reference) | 1.18 (1.05-1.33) | 1.16 (0.99-1.34) | 1.34 (1.10-1.62) |  |
| **Hypertension** |  |  |  |  | 0.344 |
| No | 1.00 (reference) | 1.08 (1.00-1.17) | 1.20 (1.09-1.32) | 1.34 (1.19-1.52) |  |
| Yes | 1.00 (reference) | 1.09 (1.01-1.17) | 1.13 (1.03-1.24) | 1.36 (1.20-1.54) |  |

Abbreviations: BMI, body mass index; CVD, cardiovascular disease.

Analyses were performed using fully adjusted Cox proportional hazards regression models, age, sex, ethnicity, Townsend deprivation index, education, body mass index, smoking status, alcohol intake frequency, sleep duration, physical activity, hypertension, dyslipidemia, diabetes, cardiovascular disease, and aspirin use.

**Supplementary Table 8 Stratified analyses for the association between frequency of adding salt to foods and the incidence of carotid artery stenosis.**

| **Subgroup** | **Frequency of adding salt to foods, HR (95% CI)** | | | | ***P* for interaction** |
| --- | --- | --- | --- | --- | --- |
|  | **Never/rarely** | **Sometimes** | **Usually** | **Always** |  |
| **Age** |  |  |  |  | **0.036** |
| < 60 years | 1.00 (reference) | 1.08 (0.98-1.18) | 1.08 (0.95-1.23) | 1.35 (1.15-1.57) |  |
| ≥ 60 years | 1.00 (reference) | 1.07 (1.01-1.14) | 1.05 (0.97-1.14) | 1.19 (1.06-1.33) |  |
| **Sex** |  |  |  |  | 0.675 |
| Female | 1.00 (reference) | 1.04 (0.96-1.12) | 1.06 (0.94-1.18) | 1.22 (1.05-1.42) |  |
| Male | 1.00 (reference) | 1.10 (1.02-1.17) | 1.06 (0.97-1.16) | 1.28 (1.14-1.44) |  |
| **BMI** |  |  |  |  | 0.943 |
| < 30 kg/m^2^ | 1.00 (reference) | 1.06 (1.00-1.13) | 1.04 (0.96-1.13) | 1.24 (1.11-1.39) |  |
| ≥ 30 kg/m^2^ | 1.00 (reference) | 1.09 (1.00-1.19) | 1.08 (0.96-1.22) | 1.26 (1.08-1.48) |  |
| **Smoking status** |  |  |  |  | 0.665 |
| Non-current | 1.00 (reference) | 1.06 (1.01-1.12) | 1.07 (0.99-1.15) | 1.25 (1.12-1.39) |  |
| Current | 1.00 (reference) | 1.13 (0.98-1.29) | 1.01 (0.86-1.20) | 1.25 (1.04-1.50) |  |
| **Alcohol intake frequency** |  |  |  |  | 0.511 |
| < 3 times/week | 1.00 (reference) | 1.08 (1.01-1.16) | 1.06 (0.97-1.16) | 1.19 (1.05-1.35) |  |
| ≥ 3 times/week | 1.00 (reference) | 1.06 (0.98-1.14) | 1.05 (0.95-1.16) | 1.34 (1.16-1.54) |  |
| **Dyslipidemia** |  |  |  |  | 0.216 |
| No | 1.00 (reference) | 1.04 (0.98-1.11) | 1.04 (0.96-1.13) | 1.28 (1.15-1.42) |  |
| Yes | 1.00 (reference) | 1.16 (1.05-1.27) | 1.11 (0.97-1.25) | 1.20 (1.01-1.43) |  |
| **Diabetes** |  |  |  |  | 0.694 |
| No | 1.00 (reference) | 1.06 (1.01-1.12) | 1.06 (0.98-1.13) | 1.25 (1.13-1.38) |  |
| Yes | 1.00 (reference) | 1.18 (0.98-1.41) | 1.04 (0.81-1.35) | 1.32 (0.95-1.82) |  |
| **CVD** |  |  |  |  | **0.033** |
| No | 1.00 (reference) | 1.08 (1.02-1.14) | 1.09 (1.00-1.17) | 1.31 (1.18-1.45) |  |
| Yes | 1.00 (reference) | 1.04 (0.94-1.16) | 0.96 (0.83-1.12) | 1.07 (0.88-1.29) |  |
| **Hypertension** |  |  |  |  | 0.186 |
| No | 1.00 (reference) | 1.08 (1.01-1.16) | 1.09 (0.99-1.20) | 1.32 (1.16-1.49) |  |
| Yes | 1.00 (reference) | 1.07 (0.99-1.15) | 1.02 (0.92-1.13) | 1.18 (1.03-1.35) |  |

Abbreviations: BMI, body mass index; CVD, cardiovascular disease.

Analyses were performed using fully adjusted Cox proportional hazards regression models, age, sex, ethnicity, Townsend deprivation index, education, body mass index, smoking status, alcohol intake frequency, sleep duration, physical activity, hypertension, dyslipidemia, diabetes, cardiovascular disease, and aspirin use.

**Supplementary Table 9 Sensitivity analyses for the association between frequency of adding salt to foods and risk of peripheral artery disease.**

| **Models** | **Frequency of adding salt to foods** | | | | |
| --- | --- | --- | --- | --- | --- |
|  | **Never/rarely** | **Sometimes** | **Usually** | **Always** | ***P* for trend** |
| Excluding participants with missing data on covariates | 1.00 (reference) | 1.10 (1.03-1.18) | 1.21 (1.11-1.31) | 1.34 (1.20-1.51) | <0.001 |
| Excluding participants with CVD at baseline | 1.00 (reference) | 1.06 (1.00-1.13) | 1.16 (1.08-1.26) | 1.36 (1.23-1.50) | <0.001 |
| Excluding participants who developed PAD < 2 years after recruitment | 1.00 (reference) | 1.05 (0.99-1.11) | 1.15 (1.07-1.24) | 1.33 (1.22-1.46) | <0.001 |
| Excluding participants who had changed their diet in last 5 years | 1.00 (reference) | 1.06 (0.98-1.14) | 1.10 (1.00-1.21) | 1.35 (1.20-1.51) | <0.001 |
| Further adjusted for Model 3 | 1.00 (reference) | 1.08 (1.02-1.13) | 1.16 (1.09-1.24) | 1.36 (1.25-1.49) | <0.001 |

Abbreviations: CVD, cardiovascular disease; PAD, peripheral artery disease.

Analyses were performed using fully adjusted Cox proportional hazards regression models, age, sex, ethnicity, Townsend deprivation index, education, body mass index, smoking status, alcohol intake frequency, sleep duration, physical activity, hypertension, dyslipidemia, diabetes, cardiovascular disease, and aspirin use.

Model 3 further adjusted for eGFR, diet score, Atrial fibrillation, chronic kidney disease, anti-hypertensive medication, lipid-lowering medication, anti-diabetic medication.

**Supplementary Table 10 Sensitivity analyses for the association between frequency of adding salt to foods and risk of carotid artery stenosis.**

| **Models** | **Frequency of adding salt to foods** | | | | |
| --- | --- | --- | --- | --- | --- |
|  | **Never/rarely** | **Sometimes** | **Usually** | **Always** | ***P* for trend** |
| Excluding participants with missing data on covariates | 1.00 (reference) | 1.09 (1.02-1.16) | 1.08 (1.00-1.18) | 1.35 (1.21-1.51) | <0.001 |
| Excluding participants with CVD at baseline | 1.00 (reference) | 1.08 (1.02-1.14) | 1.09 (1.00-1.17) | 1.31 (1.18-1.45) | <0.001 |
| Excluding participants who developed CAS < 2 years after recruitment | 1.00 (reference) | 1.08 (1.02-1.14) | 1.16 (1.08-1.24) | 1.35 (1.23-1.47) | <0.001 |
| Excluding participants who had changed their diet in last 5 years | 1.00 (reference) | 1.07 (1.00-1.15) | 1.05 (0.96-1.16) | 1.36 (1.21-1.53) | <0.001 |
| Further adjusted for Model 3 | 1.00 (reference) | 1.07 (1.01-1.12) | 1.05 (0.98-1.13) | 1.25 (1.14-1.37) | <0.001 |

Abbreviations: CVD, cardiovascular disease; CAS, carotid artery stenosis.

Analyses were performed using fully adjusted Cox proportional hazards regression models, age, sex, ethnicity, Townsend deprivation index, education, body mass index, smoking status, alcohol intake frequency, sleep duration, physical activity, hypertension, dyslipidemia, diabetes, cardiovascular disease, and aspirin use.

Model 3 further adjusted for eGFR, diet score, Atrial fibrillation, chronic kidney disease, anti-hypertensive medication, lipid-lowering medication, anti-diabetic medication.

**Supplementary Table 11 Plasma metabolites selected by the LASSO regression to create metabolic signature score for frequency of adding salt to foods (always vs. never/rarely).**

| **Metabolites** | **Group** | **Weights** |
| --- | --- | --- |
| 3-Hydroxybutyrate | Ketone bodies | 0.0229 |
| Acetate | Ketone bodies | -0.1218 |
| Acetoacetate | Ketone bodies | 0.0045 |
| Acetone | Ketone bodies | 0.0245 |
| Albumin | Fluid balance | -0.1040 |
| Creatinine | Fluid balance | 0.0127 |
| Citrate | Glycolysis related metabolites | -0.0877 |
| Glycoprotein Acetyls | Inflammation | 0.1242 |
| Linoleic Acid to Total Fatty Acids percentage | Fatty acids | -0.2230 |
| Monounsaturated Fatty Acids | Fatty acids | 0.0078 |
| Monounsaturated Fatty Acids to Total Fatty Acids percentage | Fatty acids | 0.0414 |
| Omega-3 Fatty Acids to Total Fatty Acids percentage | Fatty acids | -0.3179 |
| Docosahexaenoic Acid to Total Fatty Acids percentage | Fatty acids | -0.0250 |
| Average Diameter for LDL Particles | Lipoprotein particle sizes | 0.0843 |
| Cholesteryl Esters in Small HDL | Lipoprotein subclasses | 0.0058 |
| Concentration of Small HDL Particles | Lipoprotein subclasses | 0.1024 |
| Cholesterol to Total Lipids in Very Large HDL percentage | Relative lipoprotein lipid concentrations | 0.0493 |
| Cholesteryl Esters to Total Lipids in Large LDL percentage | Relative lipoprotein lipid concentrations | -0.0808 |
| Cholesteryl Esters to Total Lipids in Very Large VLDL percentage | Relative lipoprotein lipid concentrations | 0.0284 |
| Free Cholesterol to Total Lipids in IDL percentage | Relative lipoprotein lipid concentrations | -0.0123 |
| Phospholipids to Total Lipids in Medium VLDL percentage | Relative lipoprotein lipid concentrations | 0.0111 |
| Phospholipids to Total Lipids in Very Small VLDL percentage | Relative lipoprotein lipid concentrations | 0.0059 |
| Isoleucine | Amino acids | 0.0207 |
| Glutamine | Amino acids | 0.0445 |
| Spectrometer-corrected alanine | Amino acids | -0.0605 |
| Valine | Amino acids | -0.0587 |

Abbreviations: HDL, high density lipoproteins; LDL, low density lipoproteins; IDL, intermediate density lipoproteins; VLDL, very low-density lipoproteins.

**Supplementary Table 12 Association of the metabolic signature score and selected metabolites with frequency of adding salt to foods (always vs. never/rarely).**

| **Metabolites** | **Coefficients*** | ***P*** |
| --- | --- | --- |
| Metabolic signature score | 0.3374 | <0.001 |
| 3-Hydroxybutyrate | 0.0630 | <0.001 |
| Acetate | -0.0554 | <0.001 |
| Acetoacetate | 0.0623 | <0.001 |
| Acetone | 0.0482 | <0.001 |
| Albumin | -0.0904 | <0.001 |
| Creatinine | 0.0209 | 0.007 |
| Citrate | -0.0362 | <0.001 |
| Glycoprotein Acetyls | 0.0825 | <0.001 |
| Linoleic Acid to Total Fatty Acids percentage | -0.1031 | <0.001 |
| Monounsaturated Fatty Acids | 0.1232 | <0.001 |
| Monounsaturated Fatty Acids to Total Fatty Acids percentage | 0.1587 | <0.001 |
| Omega-3 Fatty Acids to Total Fatty Acids percentage | -0.2132 | <0.001 |
| Docosahexaenoic Acid to Total Fatty Acids percentage | -0.2138 | <0.001 |
| Average Diameter for LDL Particles | -0.0172 | 0.075 |
| Cholesteryl Esters in Small HDL | 0.0982 | <0.001 |
| Concentration of Small HDL Particles | 0.1062 | <0.001 |
| Cholesterol to Total Lipids in Very Large HDL percentage | 0.0309 | <0.001 |
| Cholesteryl Esters to Total Lipids in Large LDL percentage | -0.0156 | 0.005 |
| Cholesteryl Esters to Total Lipids in Very Large VLDL percentage | -0.0444 | <0.001 |
| Free Cholesterol to Total Lipids in IDL percentage | -0.0699 | <0.001 |
| Phospholipids to Total Lipids in Medium VLDL percentage | -0.0128 | 0.127 |
| Phospholipids to Total Lipids in Very Small VLDL percentage | 0.0096 | 0.296 |
| Isoleucine | 0.0027 | 0.778 |
| Glutamine | -0.0101 | 0.318 |
| Spectrometer-corrected alanine | -0.0617 | <0.001 |
| Valine | -0.0414 | <0.001 |

*The coefficients from multivariable linear models quantify the association between “always” adding salt to foods and changes in the metabolic signature score and individual metabolites, expressed as SD changes relative to the reference group.

**Supplementary Table 13 Mediating role of selected metabolites in the association between frequency of adding salt to foods and extracoronary vascular disease.**

| **Metabolites** | **Peripheral artery disease** | | **Carotid artery stenosis** | |
| --- | --- | --- | --- | --- |
|  | **HR 95% CI** | **PM (%)** | **HR 95% CI** | **PM (%)** |
| 3-Hydroxybutyrate | 1.08 (1.02-1.14) | 2.3 (0.3-3.4) | 0.99 (0.94-1.04) | - |
| Acetate | 0.96 (0.89-1.04) | - | 0.95 (0.89-1.02) | - |
| Acetoacetate | 1.08 (1.03-1.14) | 2.4 (0.3-4.0) | 1.00 (0.95-1.05) | - |
| Acetone | 1.06 (1.00-1.12) | 1.3 (0.3-2.8) | 0.99 (0.94-1.04) | - |
| Albumin | 0.89 (0.85-0.94) | 5.0 (2.6-9.2) | 0.92 (0.88-0.96) | 3.3 (1.9-6.9) |
| Average Diameter for LDL Particles | 0.99 (0.94-1.03) | - | 1.04 (1.00-1.09) | - |
| Cholesterol to Total Lipids in Very Large HDL percentage | 1.00 (0.95-1.06) | - | 0.95 (0.90-1.00) | - |
| Cholesteryl Esters in Small HDL | 0.91 (0.87-0.96) | - | 0.95 (0.91-1.00) | - |
| Cholesteryl Esters to Total Lipids in Large LDL percentage | 0.88 (0.81-0.96) | 0.9 (0.2-1.3) | 0.99 (0.92-1.07) | - |
| Cholesteryl Esters to Total Lipids in Very Large VLDL percentage | 0.98 (0.93-1.04) | - | 1.01 (0.96-1.06) | - |
| Citrate | 1.02 (0.97-1.07) | - | 0.94 (0.90-0.98) | 1.0 (0.2-2.6) |
| Concentration of Small HDL Particles | 0.93 (0.89-0.98) | - | 0.97 (0.92-1.01) | - |
| Creatinine | 1.08 (1.03-1.14) | 0.8 (0.3-3.6) | 1.04 (0.99-1.09) | - |
| Docosahexaenoic Acid to Total Fatty Acids percentage | 0.92 (0.87-0.97) | 8.9 (5.2-11.2) | 0.95 (0.91-1.00) | 4.5 (1.3-7.7) |
| Free Cholesterol to Total Lipids in IDL percentage | 0.98 (0.93-1.03) | - | 0.96 (0.92-1.00) | 1.2 (0.8-2.8) |
| Glutamine | 0.98 (0.93-1.02) | - | 1.00 (0.95-1.04) | - |
| Glycoprotein Acetyls | 1.24 (1.18-1.30) | 8.6 (5.5-14.5) | 1.09 (1.04-1.14) | 3.0 (2.1-5.4) |
| Isoleucine | 1.02 (0.97-1.07) | - | 1.00 (0.95-1.04) | - |
| Linoleic Acid to Total Fatty Acids percentage | 0.86 (0.82-0.90) | 7.6 (4.4-9.0) | 0.94 (0.89-0.98) | 3.0 (0.8-4.7) |
| Monounsaturated Fatty Acids | 1.08 (1.03-1.14) | 4.7 (2.6-7.7) | 1.04 (1.00-1.09) | 2.3 (0.7-4.6) |
| Monounsaturated Fatty Acids to Total Fatty Acids percentage | 1.16 (1.10-1.23) | 11.7 (5.8-22.1) | 1.08 (1.03-1.13) | 5.2 (3.7-12.9) |
| Omega-3 Fatty Acids to Total Fatty Acids percentage | 0.94 (0.89-0.98) | 7.1 (3.8-17.6) | 0.96 (0.92-1.01) | - |
| Phospholipids to Total Lipids in Medium VLDL percentage | 1.01 (0.96-1.07) | - | 1.02 (0.97-1.07) | - |
| Phospholipids to Total Lipids in Very Small VLDL percentage | 1.16 (1.11-1.22) | - | 1.08 (1.03-1.13) | - |
| Spectrometer-corrected alanine | 1.04 (0.99-1.09) | - | 0.97 (0.93-1.01) | - |
| Valine | 1.04 (0.99-1.09) | - | 0.99 (0.95-1.04) | - |

Abbreviations: PM, proportions mediated; HDL, high density lipoproteins; LDL, low density lipoproteins; IDL, intermediate density lipoproteins; VLDL, very low-density lipoproteins.

Analyses were performed using fully adjusted Cox proportional hazards regression models, age, sex, ethnicity, Townsend deprivation index, education, body mass index, smoking status, alcohol intake frequency, sleep duration, physical activity, hypertension, dyslipidemia, diabetes, CVD, and aspirin use.
